# Assessing Evidence to Guide Primary Prevention of Pathogen X

**DOI:** 10.3201/eid3206.260293

**Published:** 2026-06

**Authors:** Iris Holmes, Neil M. Vora, Emily S. Gurley, Latiffah Hassan, Wanda Markotter, James O. Lloyd-Smith, Raina K. Plowright

**Affiliations:** Cornell University College of Veterinary Medicine, Ithaca, New York, USA (I. Holmes, R.K. Plowright); School of Biological Sciences, Southern Illinois University, Carbondale, Illinois, USA (I. Holmes); Preventing Pandemics at the Source Coalition, New York, New York, USA (N.M. Vora); Johns Hopkins Bloomberg School of Public Health, Baltimore, Maryland, USA (E.S. Gurley); University of Missouri, Columbia, Missouri, USA (L. Hassan); University of Pretoria, Pretoria, South Africa (W. Markotter); University of California Los Angeles, Los Angeles, California, USA (J.O. Lloyd-Smith).

**Keywords:** spillover, pandemic, primary prevention, secondary prevention, Pathogen X, viruses, zoonoses

## Abstract

Primary prevention includes interventions that prevent the initial occurrence of disease; in the context of pandemic origins, one class of primary preventative interventions involves reducing the risk of zoonotic pathogen spillover. Pandemics are rare events, therefore data on spillover events of known pandemic pathogens are also rare. In contrast, many zoonotic viruses spill over frequently but fail to spread efficiently between humans. We consider whether insights from frequent spillovers of poorly-spreading viruses should be used to inform primary prevention strategies aimed at viruses that spill over rarely but spread well human-to-human. We propose a set of principles to steer future research and guide deployment of preventative strategies. We believe that a precautionary approach, grounded in evidence from viruses that spill over frequently, offers the most practical empirical foundation for guiding primary spillover prevention.

The COVID-19 pandemic and ongoing spillovers of avian influenza show the magnitude and urgency of threats posed by viral pandemics. Many strategies exist to mitigate those threats. In addition to well established strategies such as vaccination and therapeutics that can be employed after a pandemic pathogen begins to spread between humans, a crucial class of interventions reduce the initial risk that spillover of a virus from nonhuman animals to humans occurs (*1*). Those interventions, which are a subset of primary preventative strategies, should be key tools for reducing pandemic threats. The group of primary preventative strategies we discuss in this article target the conditions that enable spillover itself, potentially including reservoir host ecology, contact patterns between species, occupational practices, or environmental conditions that enable human exposure to zoonotic pathogens (*2*).

The key to developing evidence-based primary preventative strategies to reduce the probability of pandemic-potential virus spillover is to understand the factors governing spillover of these viruses (*3*–*5*). We are particularly concerned about understanding the spillover of a pathogen with the potential to trigger a severe global epidemic (Pathogen X) (*6*). Ideally, researchers would study how past pandemic viruses spilled over and find generalities there, but pandemics are rare occurrences (*7*,*8*) and we lack a comprehensive understanding of the conditions that enabled spillover for any past pandemic. The lack of data on spillovers of known pandemic pathogens poses a critical problem for developing primary spillover prevention strategies.

Although pandemics are rare, spillover events are not (*9*,*10*). Many zoonotic viruses spill over and are frequently detected but fail to establish sustained human-to-human transmission (*11*). For example, Puumala virus, rabies virus, and Lassa virus, among many others, are frequently-spilling zoonotic viruses that collectively account for many thousands of reported spillovers from animals to humans each year (*9*,*12*–*14*). Those frequent spillovers present an opportunity: studying them can provide insights into the ecological, sociologic, and virologic processes that drive spillover of zoonotic viruses (*3*). Data from zoonotic viruses that do not spread between humans without exceptional circumstances (basic reproduction number [R_0_] = 0) offer a distinct advantage for investigation of spillover, because every case results from a spillover event. This feature means that data on the spillover of those viruses is not confounded by cases that arise from human-to-human transmission. Viruses that exhibit limited human-to-human transmission (0<R_0_<1), such as Middle East respiratory syndrome coronavirus or Nipah virus, require additional investigatory effort to distinguish cases that arose from spillover versus cases originating from human-to-human transmission (*15*). However, those viruses can also provide a wealth of data on the spillover process, if index cases can be identified and they are comprehensively investigated (*16*).

This potential rich source of data on viral spillover raises a crucial question: do pandemic-potential viruses share spillover pathways with viruses that spill over more frequently but spread poorly? Can we assume that data on the ecological, virologic, social, or other conditions that enable the spillover of frequently spilling but poorly spreading viruses are relevant to primary pandemic prevention? To conceptualize this problem, we classified viruses along 2 dimensions (*17*): first, describing the frequency of reported spillover events from animals to humans; and second, describing the efficiency of human-to-human spread following spillover (Figure). The spillover frequency axis records the relative commonness or rarity of reported human cases arising from a zoonotic spillover. The human-to-human transmission axis captures the efficiency of onward spread among humans, as summarized by the effective reproduction number, R (or in wholly susceptible populations, R_0_) (*18*). Our placement of known pathogens on those axes comes with important caveats. First, R can vary across circumstances because of sociologic, environmental, and virologic factors; it can also vary because of population immunity. Because R can vary, placement of specific viruses is approximate, but the y-axis of the plot represents the boundary between pathogens that could possibly cause an epidemic or pandemic (viruses that spread well with R>1) and those that cannot spread well enough to sustain transmission in humans (poor spreaders with R<1) (*18*). Pathogens that cannot spread between humans (R = 0) are on the extreme left. For the spillover frequency-axis, we emphasize that the placement of viruses reflects reported spillovers, which is likely an underestimate of true spillover rates given underreporting. We can confidently assign viruses whose spillovers are frequently reported to the frequently-spilling category, but viruses with more rarely reported spillovers could potentially be in the frequently-spilling category if spillovers are often undetected or unreported.

**Figure:**
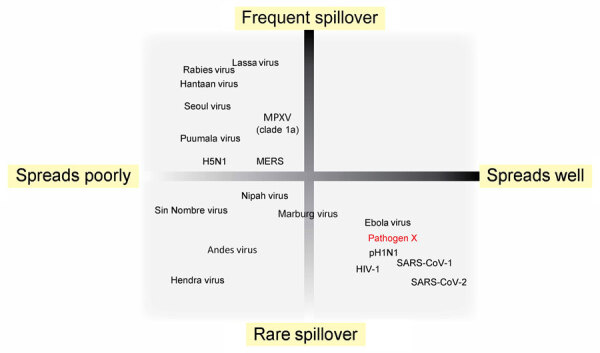
Conceptual framework for the frequency of spillover versus potential for human-to-human transmission to assess evidence to guide primary prevention of Pathogen X. Zoonotic viruses are positioned along 2 axes: spillover frequency and human-to-human spread. The y-axis line represents the boundary between viruses capable of sustained spread among humans (R>1), versus those with limited or no onward spread (R<1). The x-axis represents a relative, rather than 1-to-1, mapping of R for the example viruses. Viruses that are not known to spread human-to-human are shown at the far left of the plot. Pathogen X represents a hypothetical pandemic-capable virus and occupies the rare spillover/spreads well quadrant. MERS, Middle East respiratory syndrome; MPXV, monkeypox virus; pH1N1, pandemic H1N1.

Our framework yields 4 quadrants (Figure). Most spillover data come from viruses in the frequent spillover/spreads poorly quadrant (e.g., rabies virus, Lassa virus, monkeypox virus [MPXV], and Puumala virus), whereas spillover events in the rare spillover/spreads well quadrant are less common (e.g., pandemic influenza virus or HIV). Viruses in the frequent spillover/spreads well quadrant are likely to already be endemic in the human population. Differentiating between the frequent spillover/spreads well and rare spillover/spreads well quadrants will be challenging in practice because any further spillovers of a well spreading virus would be difficult to detect without extensive investigation of all cases. As such, our placement of viruses along this axis is somewhat tentative for viral lineages that have not undergone extensive genetic investigation. Viruses have the potential to shift quadrants or occupy multiple quadrants, depending on their ecological, sociologic, and immunologic context. For example, MPXV could not spread effectively in human populations when smallpox vaccination was widespread but has spread more as the immunologic landscape has changed. Because of the demographic turnover since mass smallpox vaccination ended, MPXV recently has caused major epidemics (*19*–*21*). Similarly, Ebola virus exhibited more sustained human-to-human transmission in the densely connected populations of West Africa than in Central Africa (*22*). In addition, changing land use practices or occupational risks could alter the rate of spillover of some viruses (*23*,*24*).

Pathogen X likely fits into the rare spillover/spreads well quadrant. Pathogen X must either spread efficiently in humans at the time of spillover or have the capacity to evolve to gain this ability (*6*). Pathogen X is rare because we have not yet seen it, so its spillover, emergence, and detection require conditions that have not yet aligned. If Pathogen X frequently spilled over, it would already be known as an emerging zoonotic pathogen or it would already be endemic within human populations (*25*); possible examples could include the 2 common cold coronaviruses that likely spilled over from bats centuries to millennia ago (*26*–*28*), or measles virus that spilled over from cattle (*29*). However, reconstructing the historic spillover rates of those viruses is not possible because any additional spillovers after their emergence were not noted in historical records. Even today, additional spillovers of those or other human-endemic viruses would not be identified without extensive investigation of viral genetics in both humans and potential reservoir species. Therefore, their placement with respect to the spillover frequency axis is unknown. Alternatively, Pathogen X could emerge when a known virus in any quadrant that undergoes genetic change (mutation, recombination, or reassortment) results in a novel variant with efficient human-to-human transmission.

To assess whether lessons from viruses in the frequent spillover/spreads poorly quadrant can be applied to the spillover process of viruses in the rare spillover/spreads well quadrant, including potential Pathogen X viruses, we should consider the factors that must align to give rise to a successful spillover event (*3*). First, for a spillover to occur, the reservoir host must overlap in space and time with human or bridging hosts and release a pathogen by some route of excretion (e.g., urine, feces, or respiratory droplets) or by direct contact with a human (e.g., hunting, butchering, animal bite). Second, for viruses not spread by direct contact, the virus might need to survive in the environment until it encounters a susceptible human or bridging host. Finally, the virus must have contact with a human via some exposure route and establish productive infection in human tissues. The key question is whether those constituent processes, which together give rise to spillover, will differ systematically between viruses that can spread efficiently among humans and those that cannot.

This question is not easily answered with current knowledge. Human-to-human spread with R>1 clearly depends on biological properties of the virus (e.g., the ability to replicate in the human host, giving rise to viral load in tissues that lead to shedding or other means of virus transfer), but also on myriad social, cultural, and environmental factors (*30*,*31*). Even when R>1, the likelihood that any given spillover event will become established and cause an epidemic or pandemic is shaped by other factors, ranging from human population connectivity to individual variation and stochasticity (*32*). Pandemic potential depends on many factors extrinsic to the virus, and possible associations with spillover rates are unclear (*25*). Yet some aspects of viral biology are correlated with both spillover and human-to-human transmission, offering hope that unifying principles might be found. For example, past comparative work has indicated that some viral traits affect both spillover risk and human-to-human transmission ability after spillover. For example, having an envelope can be correlated positively with spillover rates for a viral lineage (*33*,*34*) but negatively with human-to-human transmission ability for emerging viruses (*35*,*36*). However, many examples of closely related and structurally similar viruses that differ both in spillover frequency and subsequent human-to-human transmission dynamics exist. For example, different influenza A viruses could occupy every quadrant (Figure) (*37*,*38*). Many viruses with traits apparently conducive to both spillover and human-to-human transmission still fail to propagate within human populations, indicating that additional barriers are at play. Those could include mismatches between viral traits and host factors, immunologic constraints in human hosts, and the absence of key sociologic or environmental conditions needed for sustained spread. The complex and multiscale drivers of transmissibility are not yet sufficiently understood to enable a bottom-up, a priori analysis of our question.

We propose a phased research agenda to gain robust insights into our original question: whether studying viruses that spill over frequently but spread poorly can inform our prevention of the spillover of the next pandemic virus. The effort would begin with systems-level research to assemble and advance what is known about the ecologic, sociologic, and virologic determinants of spillover and onward transmission across a range of pathogens represented in the 4 quadrants (Figure). Using established frameworks as a guide (*3*), such data could be analyzed to compare the determinants of spillover success along an axis of R, ideally stratifying by the transmission routes and tissue tropism involved. An example research question of this type could be: What similarities and what differences exist between the pathways to spillover of Nipah virus, spread by the respiratory route in humans with R<1, and the pathways of SARS-CoV-1, also spread by the respiratory route but with R>1? How do their pathways compare to hepatitis E virus genotype 1 or 2, transmitted among humans via the fecal-oral route (*39*), or to Lassa virus or the hantaviruses, which exhibit limited or no human-to-human spread? Those investigations would require highly collaborative, multidisciplinary studies across a range of biologic scales (*16*).

This work will doubtless find many differences across systems, because those viruses and their socioecologic contexts vary greatly. However, considering the phases of the spillover process might reveal commonalities, such as 2 zoonotic systems both driven by environmental stress on their wildlife reservoir, even if virologic details or contact behaviors at the animal–human interface differ greatly. Another possibility is that insights from the literature on viral traits correlated with zoonotic risk might help to organize and understand patterns in the systems-level data (*31*,*33*,*35*). However, we emphasize that our motivating question is open-ended, and if the answer is that data from frequently-spilling viruses must be used with caution or in very specific ways to guide primary pandemic prevention, then that information is necessary. Regardless, the organizing principles we propose can help in the design of future data collection and interventions that range from pathogen-specific (e.g., vaccinating guano miners against Ebola virus) to pathogen-agnostic (e.g., regulating the wildlife trade).

Until future research can clarify factors that are unique to the spillover of rare spillover/spreads well viruses, we suggest that policy makers and public health practitioners use the precautionary approach and use all available data on spillovers to design, implement, and test primary preventative approaches. This approach recognizes the uncertainty inherent in predicting which spillover pathways might lead to the next pandemic while still enabling evidence-based interventions on modifiable risk factors across the spillover process. A salutary consequence will be the opportunity to reduce effects from frequently-spilling zoonotic viruses, which disproportionately affect the most vulnerable populations, alongside possible reduction in risk of future pandemics. In addition, this evidence-driven approach provides an opportunity to evaluate which primary prevention strategies are effective against multiple pathogens, building a generalizable toolkit for pandemic prevention.
